# A multi-centre randomised control trial of directly observed therapy to promote medication adherence in paediatric heart transplant recipients

**DOI:** 10.1017/S1047951124000775

**Published:** 2024-04-12

**Authors:** Michael O. Killian, Sonnie Mayewski, Dipankar Gupta

**Affiliations:** 1 College of Social Work, Florida State University, Tallahassee, FL, USA;; 2 College of Medicine, Florida State University, Tallahassee, FL, USA; 3 Pediatrics, Congenital Heart Center, University of Florida, Gainesville, FL, USA

**Keywords:** Paediatric heart transplantation, medication adherence, mHealth, adolescent

## Abstract

Medication non-adherence remains a significant challenge for adolescent heart transplant recipients. Building on the success of a pilot intervention study, herein we describe the protocol for a follow-up randomised control trial using mobile video directly observed therapy, featuring several innovations, to promote medication adherence in a multi-centre sample of adolescent heart transplant patients.

Adolescent patients continue to experience challenges with medication adherence following heart transplantation^1^ leading to greater rates of late acute rejection and hospitalisations.^1,2^ While higher risk of non-adherence is well documented in adolescents, efforts to improve medication adherence have had limited success.^3^ A recent pilot study of mobile directly observed therapy intervention with a sample of adolescent heart transplant recipients^4^ generated initial evidence of feasibility, patient acceptability, and demonstrated improvement in immune-suppressant medication adherence and post-transplant outcomes.^5,6^ This subsequent brief report focusses on the protocol of our follow-up and scaled randomised control trial extending this area of research to multiple transplant centres nationally.

## Mobile video directly observed therapy

A mobile video directly observed therapy mHealth application developed by Scene Health Inc. offers patients direct tracking of dose-by-dose medication-taking behaviours and interpersonal communication and support from their transplant team.^4,5^ Within the application, patients record and submit videos of themselves taking medication. Nurses review these videos asynchronously as evidence medication was correctly taken. Patients can report any symptoms of illness or medication side effects, and reviewing nurses may escalate administration problems or patient health and safety concerns to the patient’s transplant care provider.

This mHealth application provides instant feedback and dose tracking for the patient while providing the transplant team directly observed behaviour. Most importantly, the application is designed to foster communication and rapport-building with the patient. Interpersonal support and engagement were reported as valued by patients and used by nurses to offer support, encouragement, build rapport, answer medically related questions, offer patient education, and offer functional support about the therapy.^6^ Importantly, this significant rapport and interpersonal support was developed with the reviewing nurses who were previously unknown to the patients.^5,6^

Recent advancements in the mHealth application build on trends and innovations within mHealth approaches promoting adherence. A newly implemented “video-back” feature gives reviewing nurses the additional option to send patients live or pre-recorded videos, a further avenue for developing rapport and interpersonal support. Pre-recorded videos may be from the patient’s own transplant team members or other supportive individuals (e.g., patient’s family, individuals identified as sources of support by the patient). Another key innovation is the centralised review and monitoring of all patients in the programme through one transplant centre. A singular nurse or small team may provide remote, asynchronous review and patient support for a geographically dispersed number of patients. The integration of a caregiver reminder option is another new feature designed to support behavioural change and recognises the process of gradual transition of adherence responsibility from caregiver to the patient. The mHealth application and new features are designed to address multiple, modifiable risk factors for non-adherence through actively encouraging development of organisational skills, planning, and conscientiousness around the behaviour.

## Current study and procedures

The current study builds upon the prior pilot study^4,5^ and uses similar methods to examine a 12-week asynchronous, directly observed therapy intervention with adolescent heart transplant recipients from four Southeast US transplant centres during a 2-year trial period. The current randomised control trial will involve 100 patients who are at least 6 months post-transplant and non-adherent (medication level variability index^2^ > 2.0) and otherwise medically stable. Potential participants will be screened and recruited via phone calls and outpatient clinic appointments. Consenting patients aged 10–21 years will receive pre-intervention questionnaires and subsequently randomised into either the mHealth intervention or enhanced goal-setting group intervention using a randomisation procedure stratified by transplant centre (1:1 assignment ratio).

**Figure 1.**
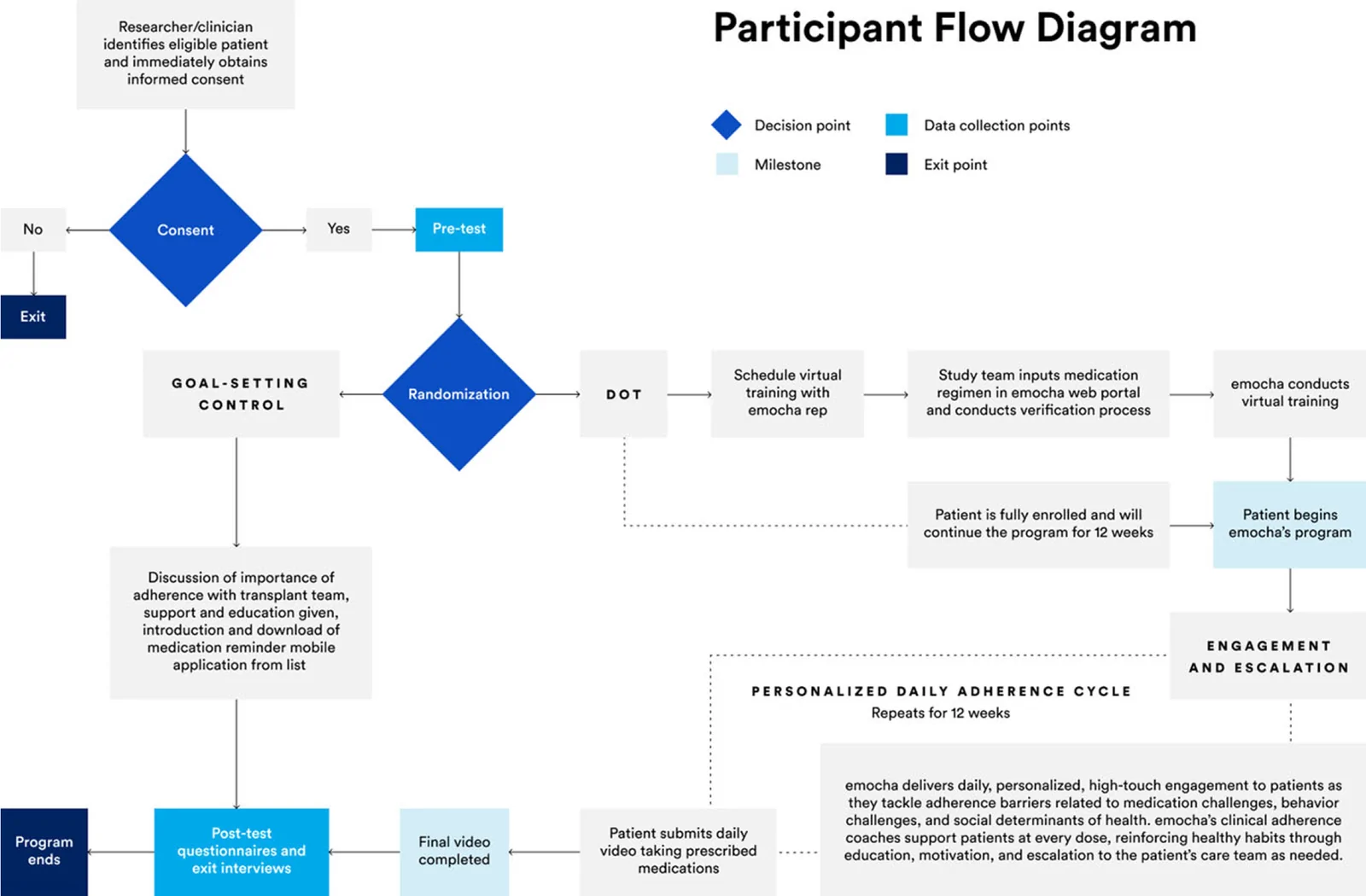
Participant flow diagram.

Patients receiving directly observed therapy via submission of mobile videos on a mHealth application will be given orientation materials and assistance with download and setup. Each patient’s care team will enter all medications into the provider-facing portion of the Scene platform. Following setup, the 12-week directly observed therapy intervention will begin and medication-taking monitored by centralised review within the application. The enhanced goal-setting condition will be a standard-of-care intervention including routine care and monitoring. Patients in this condition will receive standard patient education, materials on the importance of medication adherence, and information about standard medication reminder applications available for smart phones or similar devices. To avoid participant compensation as a motivator for adherence, all participants will receive the same compensation regardless of intervention condition (gift cards valued at 84USD, equal to 1USD per day of participation). Also, all patients will be provided with a phone and data plan to avoid selective sampling. A flow chart detailing study procedures and timeline is included in Figure 1. The study is registered with ClinicalTrials.gov.

## Measurement

Patient demographic and family characteristics will be collected from medical records and surveys (Table 1). Primary outcomes include number of successfully submitted videos reviewed as evidence of medication taking-behaviour and medication level variability index^2^ scores across the pre- and post-intervention periods. Medication adherence during the directly observed therapy intervention will be considered as the percentage of submitted and accepted patient videos demonstrating appropriate medication-taking behaviour. Long-term pre- and post-intervention adherence will be measured by medication level variability index scores calculated and from at least three patient tacrolimus (i.e., Prograf or FK506) or sirolimus blood levels from each 6 months before, during, and 6 months after participation. These scores are patient-level standard deviation values across medication blood levels with higher scores indicating greater variation in blood levels and less consistency in medication-taking. Pre- and post-intervention episodes of organ rejection and hospitalisation will also be collected as indicators of post-transplant health outcomes.

Patients and parents will receive invitations to complete online surveys for secondary outcomes measures before beginning medication level variability index and after completion of the 12-week programme. Patient self-reported and parental proxy-reported outcome measures completed pre- and post-intervention include measures of health-related quality of life,^7^ barriers to medication adherence,^8^ engagement and relationship with their transplant care team,^9^ and perceptions of quality of healthcare received.^10^ Patient engagement with the application will be measured using system usage data (e.g., amount, frequency, duration, and depth of use) and self-reports of user satisfaction.

Assuming 100 patients complete the 12-week intervention, the study is sufficiently powered (.80) to detect a 26.9% between-groups difference in post-intervention medication level variability index values as statistically significant. Evenly distributed between groups, and a rejection level of α = 0.05, the study will be able to detect a small effect size of Cohen’s f = 0.245 as statistically significant (power = .80) for secondary outcomes. We will use an intent-to-treat analytic approach to protect against bias introduced by participant attrition when estimating the effect of treatment group. We will examine pre-intervention differences between the directly observed therapy and control groups to determine the success of randomisation to generate comparable groups on observed characteristics. If necessary, sensitivity analyses will be conducted to determine the impact of baseline group imbalance by treating these factors as covariates in the modelling to adjust for their influence on post-intervention outcomes.

## Discussion and conclusion

Promising results from the pilot study^4–6^ led to this more robust investigation of mobile directly observed therapy among adolescent heart transplant recipients and translation of these efforts into clinical care settings. The current study builds on the prior work and furthers mHealth intervention efforts by integrating technological advances within the investigation. Improved video feedback capabilities and centralised review of patients will aid in patient engagement, efforts to improve this important health behaviour, and translation of this intervention into care of high-risk patients.

## Figures and Tables

**Table 1.** Study measures

Domain	Measures	Time Frame	Reporter
Patient and family characteristics	Demographics	Baseline	Medical records
	Medical Information	Baseline	
Medication adherence	Number of observed doses	Daily	DOT app and medical records
	Medication Level Variability Index^2^	≥12 months prior to enrollment, during intervention phase, and for 6 months post-intervention	
Medical outcomes	Late Acute Rejection	6 months post-intervention	Medical records
	Dates and lengths of hospitalisation(s)	6 months post-intervention	
Engagement with care team in DOT	Patient Activation Measure^8^	Pretest, posttest	Patient self-report
	Patient-Provider Interaction Scale^9^	Pretest, posttest	
Quality of healthcare and care coordination	Patient Assessment of Chronic Illness Care^10^	Pretest, posttest	Patient self- and parental proxy-report
App engagement	System usage data and self-report of user satisfaction	Pretest, posttest	Patient self- and parental proxy-report
Disease-specific health-related quality of life	PedsQL 3.0 Transplant Module^6^	Pretest, posttest	Patient self- and parental proxy-report
Barriers to adherence	Adolescent Medication Barriers Scale^7^	Pretest, posttest	Patient self- and parental proxy-report
	Parent Medication Barriers Scale^7^	Pretest, posttest	
